# Hybrid Multitask Learning Reveals Sequence Features Driving Specificity in the CRISPR/Cas9 System

**DOI:** 10.3390/biom13040641

**Published:** 2023-04-03

**Authors:** Dhvani Sandip Vora, Shashank Yadav, Durai Sundar

**Affiliations:** 1 Department of Biochemical Engineering and Biotechnology, Indian Institute of Technology Delhi, Hauz Khas, New Delhi 110016, India; 2 Yardi School of Artificial Intelligence, Indian Institute of Technology Delhi, Hauz Khas, New Delhi 110016, India

**Keywords:** CRISPR/Cas9, off-target, sequence features, saliency, SHAP, CNN-LSTM

## Abstract

CRISPR/Cas9 technology is capable of precisely editing genomes and is at the heart of various scientific and medical advances in recent times. The advances in biomedical research are hindered because of the inadvertent burden on the genome when genome editors are employed—the off-target effects. Although experimental screens to detect off-targets have allowed understanding the activity of Cas9, that knowledge remains incomplete as the rules do not extrapolate well to new target sequences. Off-target prediction tools developed recently have increasingly relied on machine learning and deep learning techniques to reliably understand the complete threat of likely off-targets because the rules that drive Cas9 activity are not fully understood. In this study, we present a count-based as well as deep-learning-based approach to derive sequence features that are important in deciding on Cas9 activity at a sequence. There are two major challenges in off-target determination—the identification of a likely site of Cas9 activity and the prediction of the extent of Cas9 activity at that site. The hybrid multitask CNN–biLSTM model developed, named CRISP–RCNN, simultaneously predicts off-targets and the extent of activity on off-targets. Employing methods of integrated gradients and weighting kernels for feature importance approximation, analysis of nucleotide and position preference, and mismatch tolerance have been performed.

## 1. Introduction

Clustered regularly interspersed short palindromic repeats (CRISPR) and their associated nucleases (Cas) have revolutionized genetics by allowing easy modification of the genome as well as gene regulation. CRISPR-based genomic perturbations mainly fall into two categories—Cas9-mediated knockout and Cas9-mediated activation or inhibition. Cas9-mediated knockout is carried out by the Cas9 nuclease directed to the target site by the guide RNA, creating a double-stranded break resulting in loss of function [1,2,3]. CRISPR inhibition/activation, on the other hand, employs catalytically inactive Cas9, enabling localization of an effector domain to repress or activate transcription of the target gene without inducing heritable changes to DNA [4,5].

The optimal design of the single guide RNA (sgRNA) is paramount to the efficiency of a CRISPR-based experiment. Bound to Cas9, the gRNA contains a spacer sequencer complementary to the target DNA (“protospacer”) and various loops to which Cas9 binds. It has, however, been reported in multiple studies that Cas9 acts at sequences similar to the target sequence as well, resulting in unwanted off-target effects [6,7]. The requirement of an NGG consensus PAM has been established for the recognition and cleavage of target DNA. Studies in the past have also revealed that the sequence composition of the 3′ end of the spacer and downstream sequence of the DNA contribute to efficiency; however, the position-specific effect of various spacer DNA bases is not yet known [8,9].

Despite multiple experimental and computational studies, the sequence determinants of guide RNA affinity, and in turn Cas9 efficiency, remain to be elucidated and checked for reproducibility. As the design of an efficient sgRNA is a major hurdle in the application of CRISPR/Cas9 for various research and medical purposes, it is critical to identify sequence features that play a pivotal role in influencing the activity of Cas9. Here, we report a count-based analysis of experimental off-targets to determine the preferences of bases and a trend in mismatch tolerance across the length of the sgRNA.

Recently, deep learning (DL) has been applied to high-throughput methods, which dramatically transformed our understanding of biology. In the context of understanding CRISPR activity, several DL-based approaches based on convolutional neural networks (CNNs) have been proposed—DeepCRISPR [10], DeepSpCas9 [11], CnnCrispr [12], and DL-CRISPR [13]. CNNs leverage the power of spatially invariant local receptive fields to find patterns in the input data. This approach is identical to position-weighted motif scans across the input genomic sequence [14]. Though the CNN layers have the potential to extract local signals, they are unable to extract sequential information. Hence, recurrent neural networks (RNNs) have been used to extract sequential information and hybrid models having a CNN–RNN setup have been proposed, such as C-RNNCrispr [15].

All of the methods and algorithms reported so far have been for either off-target classification or prediction of the extent of activity by regression. Here, for the first time, a novel multi-task prediction model is reported that can simultaneously identify experimentally likely off-targets by classification and predict the extent of activity by regression. A major focus of the study is to derive the contribution of features to the model output, in other words, the feature importance. The importance of the various components of the input, which is the nucleotide and position information, in this case, will allow insights into position-specific tolerances and preferences. Predictive neural network models have been trained on experimental datasets to obtain generalizable position-specific preference information. The occurrence of mismatches at each position and the propensity for various nucleotides to tolerate mismatches have been recorded for data obtained from various experiments that measure Cas9 off-target effects.

Additionally, a hybrid multitask neural network, CRISP–RCNN, consisting of convolutional and bidirectional long-short term memory layers, has been trained on experimentally validated off-target sequences. The negative set consists of similar sequences in the genome adjacent to a PAM motif, but are not acted upon by Cas9, derived using the CRISPcut method [16]. A multitask learning model was trained to simultaneously allow classification of the positive and negative off-targets and prediction of the extent of Cas9 activity on the positive off-targets. The features learned by the models that influenced the output were analysed by utilizing methods such as smoothed saliency [17] and gradient-based SHAP to determine the important regions of the sequence that contribute to the prediction, and in turn, may shed light on the factors that influence Cas9 activity.

## 2. Materials and Methods

### 2.1. Data Assembly

#### 2.1.1. Positive Off-Target Data Source

The dataset for the mismatch occurrence analysis was assembled and modified from the published dataset obtained by the unbiased genome-wide CRISPR/Cas9 off-target detection method CIRCLE-seq [18]. The sequences, along with the read count, were extracted to perform a count-based analysis to determine mismatch propensity. The same dataset was employed as the training set to develop the multitask deep learning model. Cleavage frequency-weighted analysis was performed by generating a corresponding dataset with the sequences represented in proportion to the experimental read count, which was generated using a Python script.

#### 2.1.2. Negative Off-Target Data Source

The sequence dataset required for training the classification mode of the multitask model was obtained using the off-target prediction method CRISPcut [16]. The off-targets obtained from CRISPcut were checked for duplicates and positive off-target sequences were eliminated from the negative set. Recent reports indicate that Cas9 tolerates DNA/RNA bulges as well, apart from mismatches, which are represented as gaps in terms of sequence alignment. To account for the bulges, the predicted off-targets were aligned using the pairwise2 module of biopython tools [19,20]. Pairwise alignment using a dynamic programming algorithm allows adjusting the parameters to obtain results close to those observed in experiments. The optimum parameters were obtained by testing various combinations simultaneously, then checking the number of allowed mismatches and gaps while retaining as many negative off-target sequences as possible.

#### 2.1.3. sgRNA-Target Sequence Encoding

As input for the multitask convolutional neural network prediction model, the sequences were one-hot encoded and converted to images. Front padding of ‘N’ base was added to equalize the length of the sequences after accommodating the gaps. The scheme used to convert an example sequence to an input image can be understood from Supplementary Figure S1. As the Cas9 protein tolerates mismatches between the guide RNA and DNA, two guide RNAs may have some similar off-targets depending on the number of mismatches tolerated. The sequence of targets is relevant to the off-target prediction, and thus the intended target DNA, perfectly complementary to the gRNA, is also included in the prediction pipeline along with the off-target sequences for each guide RNA.

The intended target DNA sequence information, the on-target, is supplied with all possible sequences in the genome that could be potential off-targets. If the off-target is detected in any of the experimental methods mentioned earlier, it is a positive off-target. If a similar sequence is present in the genome, but no experimental proof of Cas9 activity is available at that site, it is labelled as a negative off-target.

### 2.2. Mismatch and Bulge Propensity Analysis

The number and position of mismatches were counted along the length of the guide RNA by implementing a Python script. The corresponding nucleotide at that position in the target sequence is simultaneously noted. The script was also employed to count the number and position of gaps in the sequences, indicating a DNA/RNA bulge, while noting the corresponding target nucleotide. The results from the script were plotted as a percentage of the total to comprehend the trends in mismatch and bulge tolerance.

### 2.3. Deep Learning Techniques, Model Evaluation, and Feature Importance

#### 2.3.1. Model Architecture

**Figure 1:**
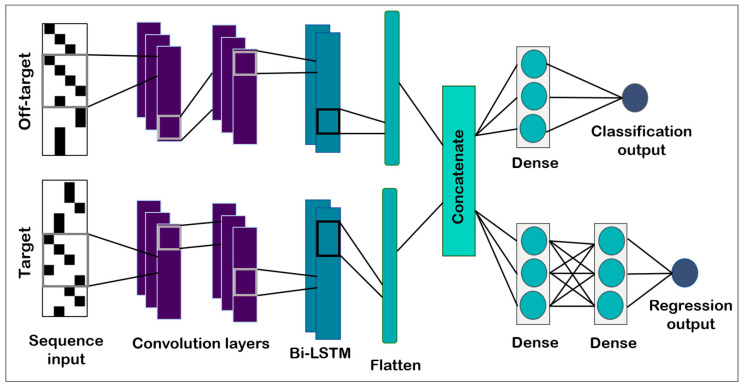
**CRISP–RCNN model architecture.** Representative target and off-target sequences are depicted as input for the model. The input is fed into convolution layers, followed by an LSTM layer. The two branches are then concatenated and fed into two separate but fully connected dense layers, which perform the classification and regression tasks.

A hybrid CNN–bidirectional LSTM network was trained; the architecture is depicted as a schematic in Figure 1 and a detailed representation is included as Supplementary Figure S6. Similar to a Siamese network, the network contained two identical sister networks taking input from the off-target and target images, respectively [21,22]. Each sister network consisted of two CNN–batch normalization blocks in tandem, feeding their output to a bidirectional LSTM layer. The sister networks were then concatenated. The output of the concatenation layer was split and fed into two separate but fully connected dense layer blocks, one for each of the classification and regression tasks. The model architecture represented a hard-sharing multitask learning model with the advantage of reducing overfitting [23]. The size of the layers was optimized using grid search [24].

#### 2.3.2. Model Training

The dataset contained 153,924 samples and was split into training/validation/testing sets in the ratio of 80:10:10. Out of the 153,924 samples, the negative off-target and positive off-target datasets contained 146,734 and 7190 samples, respectively, which led to a data imbalance of approximately 20:1. To overcome this problem, we used the ‘class_weights’ feature of keras model fitter, which balances the contribution of the minority class and the majority class to the model’s overall loss [25]. The classification and regression task models were first trained separately for different architectures on the dataset to determine the range of the losses. The losses were then combined after multiplying the regression loss by a constant factor of 2 × 10^−4^ to bring both losses to the same scale. The classification and regression losses were then added to give the final loss function, considering the model is a multitask network with hard parameter sharing.

The model encountered overfitting initially, which was later tackled by utilizing L2 regularization (optimum L2 value = 5 × 10^−4^) in the fully connected dense layers. We used the Adam Optimizer [26] initialized with a learning rate of 10^−4^, and the learning rate was halved whenever the validation loss did not decrease in the previous five epochs. We trained the different architectures on an Nvidia Titan Black GPU and evaluated the model five times with different splits of the training and validation sets and assessed the average distribution of the evaluation metrics. The best-performing CNN–biLSTM model was selected for further analysis.

#### 2.3.3. Evaluation Metrics

The model performs two tasks: (a) classification, predicting positive and negative off-targets; and (b) regression, predicting the extent of Cas9 activity at the positive off-targets. Hence, these two tasks were evaluated separately based on different performance metrics. The classification performance was tested on the basis of the following.

Area under the precision–recall curve is one among the standard criteria for classification performance evaluation. Precision measures how many of the predicted positives are correct, while recall measures how many actual positives are captured by the model. These measures are defined as follows:

$$Recall=\frac{TP}{TP+TN}$$

$$Precision=\frac{TP}{TP+FP}$$

where TP stands for true positives, TN stands for true negatives, and FP denotes false positives.

While selecting the optimal model for the purpose of classifying the off-targets, especially because the dataset is imbalanced and the positive class is the minority class, the overall recall as well as minority class recall were considered. Additionally, the Matthew’s correlation coefficient (MCC), which is known to be a reliable metric in binary classification of unbalanced datasets, was also compared among the candidate models [27].

$$MCC=\frac{TP*TN-FP*FN}{\sqrt{\left(TP+FP\right)\left(TP+FN\right)\left(TN+FP\right)\left(TN+FN\right)}}$$

The F1-MCC plot was generated to evaluate the model performance, considering the imbalance in the data. The F1-MCC curves allow the integration of many aspects of performance over multiple classification thresholds [28].

The performance of the prediction model for the regression task was measured by the coefficient of determination (R^2^). The R^2^ measures the goodness-of-fit between the predicted values and actual values of the datapoints.

#### 2.3.4. Identification of Important Sequence Features

To interpret CNN-based models, pixel attribution methods were used to locate regions in the input image that have a greater influence in deciding the model output. One such method is gradient-based saliency, which calculates the gradient of the model output with respect to input pixels and assigns importance scores to individual pixels [29]. Smoothened saliency advances the saliency approach by sampling similar images with some added noise. For each image in the samples, the saliency was calculated and the resulting saliency maps were averaged [17]. Though gradient-based saliency maps act as a great baseline, they suffer from gradient discontinuity and saturation because the negative gradients get zeroed out because of the non-linear activation layer [30]. Hence, gradient saliency methods fail at highlighting the features that contribute negatively to the model output. A gradient integration approach was used to mitigate the gradient discontinuity and saturation problem [31]. The gradients were integrated as the inputs are scaled from a reference value to the actual input value found in the dataset. A similar approach named DeepLIFT [30] computes the importance scores based on the difference between the user-defined reference input and actual inputs to the model. The positive and negative contributions were treated separately and shown to be closely connected with game-theory-based Shapely values. The ‘tf-keras-vis v0.6.2’ Python library was used to calculate the smoothened saliency and, for integrated gradient-based analysis, the ‘shap v0.39.0’ Python library was implemented.

## 3. Results

### 3.1. Data Assembly and Preparation

A total of 153,924 off-target sequences—both positive and negative—corresponding to 10 unique target sites were employed for analysis and model training. The positive dataset consists of experimentally determined off-target sites by the technique of circularization for in vitro reporting of cleavage effects by sequencing (CIRCLE-seq) [18]. The negative dataset consists of similar sequences found in the genome, identified using the tool CRISPcut [16], but not found to be cleaved in the CIRCLE-seq experiment. For each individual target, the read count for each off-target sequence was normalized and denoted as the output variable for the regressor. The positive off-targets and negative off-targets were labelled to be the outputs for the classifier. The sequences were aligned to include the information of DNA/RNA bulges. The cleaned dataset was one-hot encoded and converted to images to be used for model training and visualization, respectively. An example of the image encoding is shown in Supplementary Figure S1.

### 3.2. Analysis of Mismatch Occurrence and Nucleotide-Specific Mismatch Propensity

**Figure 2:**
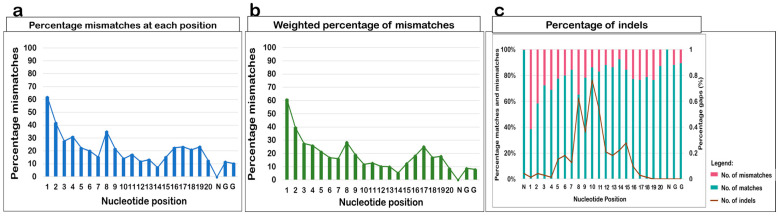
**Trend in mismatch tolerance across the length of the protospacer.** (**a**) Mismatch tolerance measured on the experimental dataset. (**b**) Mismatch tolerance weighted by experimental cleavage frequency. (**c**) Occurrence of insertions or deletions, represented as gaps, across the length of the protospacer.

The experimentally observed off-target sequences were checked for the fraction of mismatches found at each position of the 20-nucleotide guide RNA to determine the trend in mismatch tolerance for the Cas9–gRNA complex. As reported in previous studies, the number of mismatches tolerated was higher in the PAM-distal ends [32]. The overall mismatch tolerance decreases towards the PAM-proximal end. The least number of mismatches is observed in the middle region of the guide, ranging from nucleotides labelled 10–15, as demonstrated in Figure 2a. Position 14 was seen to tolerate the least number of mismatches.

A similar count-based analysis was performed again, considering the normalized cleavage frequency observed in experiments to be a weight for each sequence and, consequently, the mismatches in that sequence. The sequences would be represented fairly depending on the fraction of times it was observed to be acted upon by Cas9 in the experiment. The observed trend in mismatches remained the same, with a decrease in the tolerance towards the PAM-proximal end (Figure 2b). In both sets of analyses, the PAM region also retains the canonical NGG motif and is among the least tolerant to mismatches. Consistent with previous literature, the most common non-canonical PAM motifs are ‘NGA’ and ‘NAG’ [33]. These two PAM sites account for more than 7% out of 11% of non-canonical motifs.

**Figure 3:**
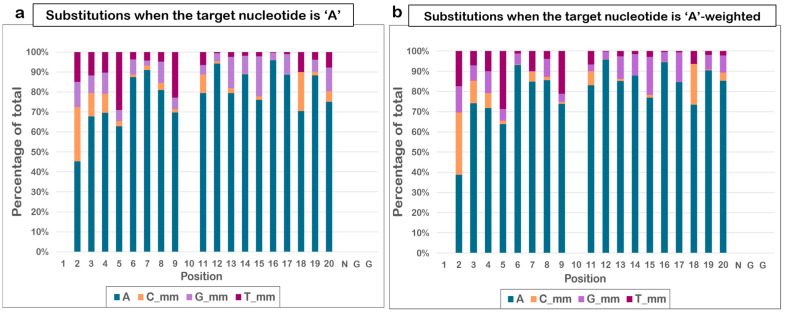
**Nucleotide-specific trend in mismatch tolerance.** (**a**) Counted for all instances when the target nucleotide is ‘A’ and (**b**) frequency-weighted trend for mismatch tolerance when ‘A’ is the target nucleotide, calculated individually for each position.

A count-based analysis was performed again for each of the four nucleotides observed at each position of the guide to determine if there is a nucleotide-specific preference for or against mismatches at certain positions of the guide. For example, considering all positions with an ‘A’ at the corresponding site in the target sequence, the mismatches at each position were noted. The fraction of the other nucleotides that contributed to the total mismatches was also noted, and the trend observed was summarized in Figure 3a. With the dataset being limited, certain positions such as 1 and 10 did not show an ‘A’ in any target sequence, thus mismatch tolerance could not be determined. For the PAM sequence, as the motif is ‘NGG’, any nucleotide present at the position of ‘N’ is not counted as a mismatch. The positions that retained the expected ‘A’ at more than 90% of all of the occurrences were 7, 12, and 16, and were said to have low mismatch tolerance.

A similar count-based analysis was performed for the other three nucleotides—‘T’, ‘G’, and ‘C’ (Supplementary Figure S2). The nucleotides that showed the least number of mismatches at each position were determined, as summarized in Table 1. Certain positions—1, 2, 10, and 17—are not included in the table because examples of all four nucleotides at these positions in the target site are unavailable in the dataset considered. Table 1 also mentions the bases most likely to be found in the case of a mismatch at each position, even when the target contains the ideal nucleotide. The most likely mismatch at each position, given the target nucleotide, will help design better tools keeping in mind the likely threats of off-targets.

Accounting for the number of times a unique sequence has been cleaved in an experiment by including it as a weight in the count-based analysis gives a more realistic understanding of the threats of the possible off-targets. Hence, the position-specific nucleotide-wise mismatch tolerance was weighted by the normalized frequency of cleavage and calculated. Table 2 highlights the key findings. The trend for the nucleotide that tolerates the least number of mismatches at each position is similar, with the exceptions of positions 7, 12, and 15. The nucleotides found to be most substituted in the off-targets also showed a similar trend to those obtained earlier.

Hence, based on mismatch occurrences observed in experiments, position–nucleotide combinations were deduced that exhibit the least tolerance to mismatches. In the rare events that mismatches were tolerated at these pairs of combinations, the presumable mismatch at that position was also identified. These can be used to collate an ideal guide with the least probable off-targets as well as the ability to identify the off-target sequences that are expected to be more of a threat than others.

On the other hand, the study also indicates the base–position combinations that tolerate the most mismatches; these would be the least favourable for the ideal guide design. The bases at each position that show the least fidelity are summarized in Supplementary Table S1.

### 3.3. Analysis of Bulge Occurrence and Nucleotide-Specific Bulge Propensity

The single RNA-guided Cas9 protein is reported to tolerate not only mismatches but DNA/RNA bulges as well, resulting in off-target effects [7,18,34]. Represented as gaps in a sequence, DNA/RNA bulges also account for a certain fraction of differences from the target site. Hence, to ascertain whether there are patterns in the tolerance of gaps in the dataset, the sequences were aligned and then analysed for mismatch tolerance at each position. The fraction of gaps tolerated across the spacer region in the context of mismatches is summarised in Figure 2c.

Most of the bulges tend to be tolerated towards the middle of the RNA–DNA hybrid, as indicated by the plot (Figure 2c). A nucleotide-wise analysis of the position and type of mismatch revealed a consistency between the results obtained for this aligned sequence dataset and the unaligned dataset. The number of gaps observed at every position for each nucleotide was also noted; the position–base pairs tolerating the most gaps are summarized in Supplementary Table S2. The trends in mismatch occurrence and nucleotide and position preferences did not show much deviation from the earlier observations. Weighting the gap counts by the experimental read count also revealed a similar pattern in the tolerance of DNA/RNA bulges (Supplementary Figure S3).

However, as the observations mentioned before were based on a single experimental technique, the reproducibility of the observations needs to be probed. There is a need for a method that is both accurate on unseen data and can elucidate the importance of a nucleotide at a specific position. Therefore, a deep-learning-based approach was adopted to predict Cas9 activity at off-target sequences while retaining information about the contribution of the position and sequence features to the model output.

### 3.4. Predictive Performance of the CRISP–RCNN Model

**Figure 4:**
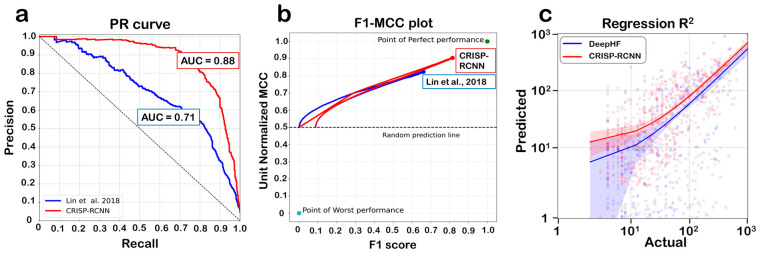
**CRISP–RCNN prediction performance.** (**a**) The precision–recall (PR) curve for CRISP–RCNN has an area under the curve (AUC) of 0.88, and performs better than the other dual-sequence-based classifier in the comparison, which has an AUC of 0.71, reported by Lin et al., 2018 [35]. (**b**) The F1-MCC curve indicating robust performance even on the imbalanced dataset; the points indicating the best- and worst-possible prediction performance are also highlighted. In plots (a,b), the dashed line indicates random prediction, i.e., 50% accuracy. (**c**) The R^2^ value for the CRISP–RCNN regressor is 0.71, showing better performance than a previous guide RNA prediction model built on sequence and physicochemical parameters, which shows an R^2^ value of 0.49 [36].

Various neural networks were trained on the target and off-target sequences to explore the nucleotides and positions deemed important for off-target activity prediction. Convolutional neural nets (CNNs), CNN coupled with a long short-term memory (LSTM) layer, and CNNs with a bidirectional LSTM layer were optimized and tested. The performance of the multiple architectures tested is summarized in Supplementary Figure S4. The best-performing model was a convolutional network with a bi-LSTM layer, referred to henceforth as CRISP–RCNN. The negative dataset consisted of sequences similar to the target sequences, with up to nine mismatches and gaps, but not cleaved by Cas9 in experiments. CRISP–RCNN performed the classification of positive and negative off-targets with an MCC of 0.80, while other classification performance metrics are summarized in Figure 4 and Table 3. The CRISP–RCNN model also simultaneously predicted the extent of Cas9 activity on the target site with an R^2^ value of 0.71 when evaluated on the held-out test dataset.

When compared against the performances of previously reported methods for performing similar tasks individually, that is, the classification performance against an off-target classifier and the regression performance against a guide RNA activity prediction model, CRISP–RCNN is shown to outperform both (Figure 4) [35,36].

### 3.5. Activation Maps

The representations of the weights in the layers of a CNN before performing the prediction task are called activation maps (AMs). AMs for a specific class would indicate the discriminative regions (in this case, nucleotide and the position of nucleotides) that the deep learning network deems critical for identification with that class [37]. AMs were implemented to interpret the feature importance in the images containing DNA sequence information in the context of understanding and improving CRISPR/Cas9 activity. The on-targets, positive off-targets, and negative off-target sequences would each generate unique AMs, highlighting the distinguishing features.

**Figure 5:**
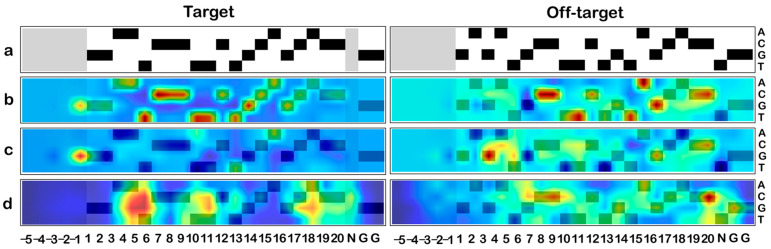
**Activation maps (AMs).** (**a**) The FANCF locus target and representative positive off-target sequences are considered. The sequence is numbered from the PAM-distal end towards the ‘NGG’ PAM site. The N-padding is numbered in negative. (**b**) DeepSHAP, (**c**) gradient explainer (GradExp), and (**d**) smoothened saliency maps are depicted. The regions marked in red indicate a positive contribution and the regions marked in blue indicate a negative contribution for (**b**,**c**). In the case of smoothened saliency (**d**), only regions that contribute to the model are marked in red, regardless of a positive or negative contribution.

The smoothened saliency, DeepSHAP, and gradient explainer SHAP (GradExp) were employed to generate AMs of the various sequences, as detailed in Section 2. The three algorithms helped visualize the models to focus on mismatches and gaps when present. DeepSHAP and GradExp highlighted the gaps and mismatches in blue, indicating a negative impact of their presence on the model’s output decision. In case of mismatches as well as gaps, the spot of the expected nucleotide is accentuated (Figure 5). In the case of the NGG PAM, the trend observed indicates a preference against the presence of a GGG PAM, which has also been established in the literature [33].

The model could successfully distinguish between the negative impact of mismatches or gaps highlighted in blue and the positive impact of features highlighted in red. The residues and positions that drive model output in the positive direction, classifying to the positive class or predicting a higher regression score, are indicated by the algorithms in shades of yellow, orange, and red. The importance of the individual bases at each position was probed further by generating average AMs across the datasets.

### 3.6. Average AMs’ Feature Importance

**Figure 6:**
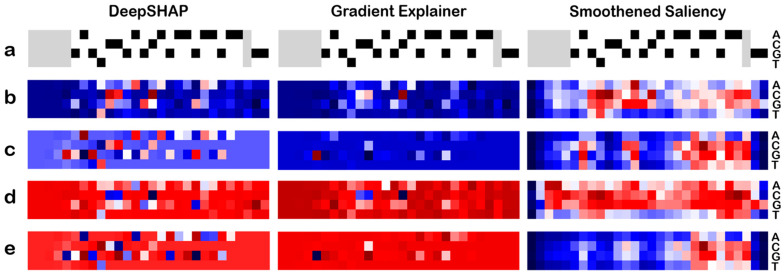
**Average activation maps.** EMX1 locus is shown as a representative example. (**a**) The EMX1 target sequence. Average activation maps of the (**b**) positive off-target sequences, (**c**) positive class reference target sequences, (**d**) negative off-target sequences, and (**e**) negative class reference target sequences.

AMs generated for multiple off-target sets of both positive and negative classes were averaged to obtain an overview of the bases and positions that were consistently marked as important across the datasets. The average saliency, DeepSHAP, and GradExp maps were generated for a single target along with both the positive and negative off-targets individually. As an example, the EMX1 target sequence was considered (Figure 6). The average saliency maps generated for the positive off-target set along with the reference target sequence are shown in Figure 6—most target bases and positions are seen to be marked similarly in both positive and negative classes, indicating a similar importance of the target sequence in both datasets. However, the DeepSHAP and GradExp average AMs indicate a similar magnitude but opposite importance of the nucleotides and positions of the target sequence in the positive class against the negative class. Although a particular base may be considered important at the same position in both classes, in the positive class, the most importance is given to bases that result in classification in the opposite class, as seen in Figure 6. Hence, most bases are marked in shades of blue, indicating a negative impact on class assignment. Although the pattern of importance in the negative class target reference sequence is similar, the bases are marked in red, indicating a positive score. The target sequence average AMs show an importance pattern mimicking the target sequence.

In the example considered, a clear preference for the variable nucleotide in the PAM region is observed in the order of ‘A’ and ‘T’, with ‘C’ and ‘G’ being the least preferred. This observation is consistent across the other target and off-target sets considered and is in accordance with previous studies [38].

The positive off-target average saliency maps indicate important bases and positions, some of which could also be observed in the case of negative off-targets. Hence, it was important to note that the positions that were marked in one set but not in the other to derive critical features. In the case of EMX1 positive off-targets, positions 1 and 2, as well as positions 11 to 18, were seen to be mostly contributing negatively. A similar trend was noted in the average AMs generated for the other target and off-target sets. The observations seem to be reinforcing the idea that seed regions tolerate fewer to no mismatches, additionally indicating that the PAM-distal-most positions also play a role in efficacy.

The average DeepSHAP and GradExp AMs of the positive off-target sets indicated positions of mismatches that result in the sequence being classified in the opposite class. As an extrapolation, the mismatch regions marked in red associate the sequence with the positive class, i.e., mismatches that were tolerated and likely to be observed in experiments. In the example of EMX1, a match at positions 4–6 and 8–10, as well as in the seed region at 15 and 16, drive the model output to classify the sequence as a positive off-target. The matches at 5, 6, and 10 are indicated to be more important than those at the other positions. A similar trend was also observed in the sets analysed.

The negative off-target AMs averaged over multiple sequences showed many similarities in the importance patterns with the positive off-target average AMs. Among the regions uniquely crucial to the negative off-target set, as seen in the averaged saliency maps, regions 1 and 2, as well as regions 11 to 18, were found to be important. The observations of DeepSHAP and GradExp were consistent in denoting positive importance to these sites when classifying to the negative set. This trend is also consistent with the opposite denotation in the positive class AMs.

## 4. Discussion

In research and clinical applications, precise genetic manipulation is a major goal. Additionally, in the case of CRISPR/Cas9, potential off-target activity and consequent mutations hinder applications of the system. The on-target activity and off-target effects of CRISPR/Cas9 are dependent on the guide RNA sequence selection and design. Understanding the sequence features that determine Cas9 activity at any genomic locus has been a long-standing goal. Here, we present a count-based analysis of experimental data as well as a deep-learning-based feature importance estimation to investigate the position-specific and base-specific tolerances and preferences of the CRISPR/Cas9 system. The trends observed indicate a lower tolerance to mismatches towards the PAM-proximal end, concordant with the “seed” region hypothesis. The trends in preferred PAM sites were also in accordance with experimental reports that, after ‘NGG’ PAM, the preferred non-canonical PAMs are ‘NAG’ and ‘NGA’ [33,38].

The CRISP–RCNN model allows insights into the sequence feature importance while reliably predicting Cas9 activity. An advantage of such a predictor is the robustness to new target and off-target data. Analysing the average AMs of the negative and positive class target reference sequences allows insight into positions that tolerate more or fewer mismatches, leading to assignment in each class. The average AMs of the positive off-target sequences indicate the mismatches that are likely to be tolerated by the Cas9 system. The positions that were found less tolerant to mismatches were the eight positions proximal to the PAM and the two to three bases most distal to the PAM site, in accordance with recent reports [32,39]. In each case, the base preference depended on the target site sequence. In certain positions of the sequence, known to tolerate mismatches, the base that is rarely observed as a mismatch is less of a threat. Hence, at each position, knowing the least-reported bases represents important knowledge. Although the importance of the seed region has been acknowledged in multiple studies, the low mismatch tolerance of the PAM-distal end has not been extensively studied. However, molecular-dynamics-based approaches have indicated that mismatches at positions 1–3 are less likely to be tolerated, while those at positions 3–10 do not hinder Cas9 nuclease activity [39]. Hence, such knowledge can be incorporated into rule-based off-target prediction algorithms, improving target selection, leading to the efficient design of CRISPR/Cas9 experiments.

The negative class off-target sequences, when analysed by generating average AMs, suggest the mismatches that are not likely to be tolerated in an experimental set-up. Among the regions that contribute to model output, those present exclusively in the negative off-target set-based AMs obtained for the various target sequences were studied. The average AMs were compared against the count-based study. Certain base–position combinations and their tolerance to a type of mismatch were found in both approaches; for example, if a ‘C’ is present at position 5, the most likely tolerated substitution is ‘A’, seen in the average AMs for EMX1 (Figure 5) and VEGFA2 (Figure S5). However, not all trends are consistent among the two analyses, implying a need for expanding the dataset to improve the count-based study. On the other hand, the consonance of the results of the deep-learning-based approach with the previous reports that aimed at establishing rules for Cas9 activity allows confidence in the predictions of the method.

## 5. Conclusions

The CRISPR/Cas9 genome editor has allowed rapid transformation of the field of biotechnology. Understanding and controlling the undesired outcomes of a CRISPR/Cas9 experiment has been an area of focus in the recent past. The study presented aims to allow an insight into the rules that steer the activity of Cas9 based on available experimental data. The CRISP–RCNN model was implemented for simultaneous classification and regression to understand position and sequence feature importance. Hence, this study will add to the rule-based guide RNA design and target site selection for efficacious Cas9 experiment design. Similar approaches can be used for optimizing other CRISPR-associated nucleases with the goal of improving research and clinical applications.

## Figures and Tables

**Table 1:** Position–nucleotide pairs with the least mismatch tolerance.

Position	Target Nucleotide	Sequences with No Mismatches (%)	Most Substituted Nucleotide	Sequences with ‘Most Substituted’ Mismatch (%)
3	T	78.57	A	12.37
4	G	94.26	A	3.79
5	C	85.58	A	6.19
6	A	87.41	G	7.65
7	A	90.93	T	4.25
8	G	86.66	A	9.31
9	C	83.09	A	7.25
11	T	92.83	A	4.09
12	C	94.77	T	3.01
13	C	95.46	T	3.93
14	C	96.32	T	2.29
15	T	89.28	A	7.89
16	A	95.85	G	3.65
18	T	83.38	A	8.35
19	C	94.14	T	3.74
20	C	88.82	T	4.93

**Table 2:** Least mismatch-tolerant position–nucleotide pairs weighted by experimental read count (also referred to as the experimental cleavage frequency).

Position	Target Nucleotide	Sequences with No Mismatches (%)	Most Substituted Nucleotide	Sequences with ‘Most Substituted’ Mismatch (%)
3	T	76.52	A	15.31
4	G	94.52	A	3.78
5	C	89.11	T	4.77
6	A	93.03	G	5.44
7	C	88.65	T	6.52
8	G	89.52	A	7.92
9	C	86.12	A	7.89
11	T	91.67	A	5.31
12	A	95.74	G	3.91
13	C	95.53	T	3.91
14	C	97.64	T	1.83
15	C	87.29	A	10.08
16	A	94.54	G	4.95
18	T	85.48	A	8.04
19	C	97.45	T	2.03
20	C	94.16	T	3.36

**Table 3:** Performance scores for various metrics of the best-performing model (CRISP–RCNN), measured on the held-out test dataset.

Metric	Score
**Precision**	0.91
**Recall**	0.87
**F1 score**	0.89
**MCC**	0.80
**Cohen’s Kappa**	0.77

## Data Availability

All of the scripts used for data processing, model training, and feature importance analysis are available at https://github.com/TeamSundar/CRISP-RCNN.git.

## Supplementary Materials

The following supporting information can be downloaded at https://www.mdpi.com/article/10.3390/biom13040641/s1, Figure S1: Sequence to image encoding scheme; Figure S2: Mismatch tolerance across the length of the guide RNA; Figure S3: gap tolerance across the length of the guide RNA; Figure S4: Predictive performance of various neural networks tested; Figure S5: Average class activation maps for the VEGFA2 locus off-targets; Figure S6: Architecture used for the dual classification and regression tasks; Table S1: Base-position pairs that tolerate the most mismatches; Table S2: Gap tolerance across the length of the guide RNA.
