# Supplementary material for: Hybrid Multitask Learning Reveals Sequence Features Driving Specificity in the CRISPR/Cas9 System
Source: Biomolecules. 2023 Apr 3;13(4):641. doi: 10.3390/biom13040641 (PMC10135716; doi:10.3390/biom13040641)
Supplement: Supplementary file 1 [file biomolecules-13-00641-s001.zip › biomolecules-2265971-supplementary.pdf]

biomolecules

Hybrid Multitask Learning Reveals Sequence Features Driving Specificity in the CRISPR/Cas9 System

Dhvani Sandip Vora [1,†], Shashank Yadav [1,†] and Durai Sundar [1,2,*]

1 Department of Biochemical Engineering and Biotechnology, Indian Institute of Technology Delhi, Hauz Khas, New Delhi 110016, India
2 Yardi School of Artificial Intelligence, Indian Institute of Technology Delhi, Hauz Khas, New Delhi 110016, India
* Correspondence: sundar@dbeb.iitd.ac.in
† These authors contributed equally to this work.

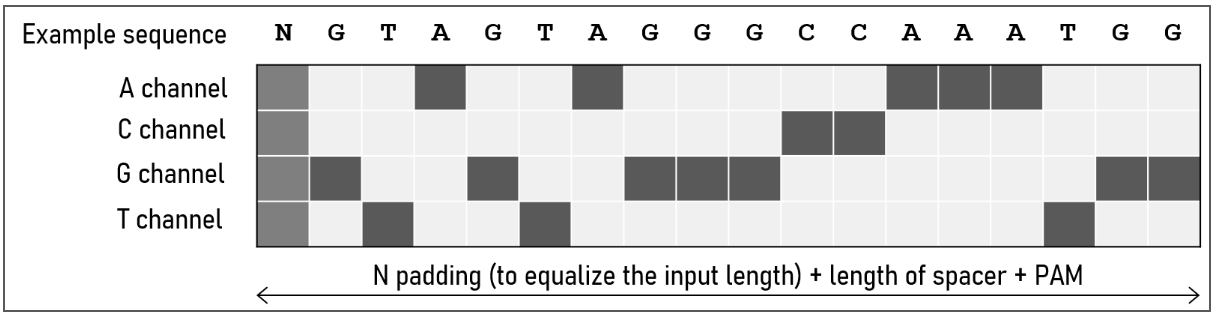

Figure S1. Sequence to image encoding scheme. The 4 channels correspond to the 4 nucleotides. Like the one-hot encoding scheme, the presence of a nucleotide marks a positive in that channel, while the other channels show no values. Here, a monochromatic scheme has been used. N-padding has been used to equalize the off-target sequence lengths. At all positions of 'N', which may be any nucleotide, a 25% intensity fill is used for all 4 channels.

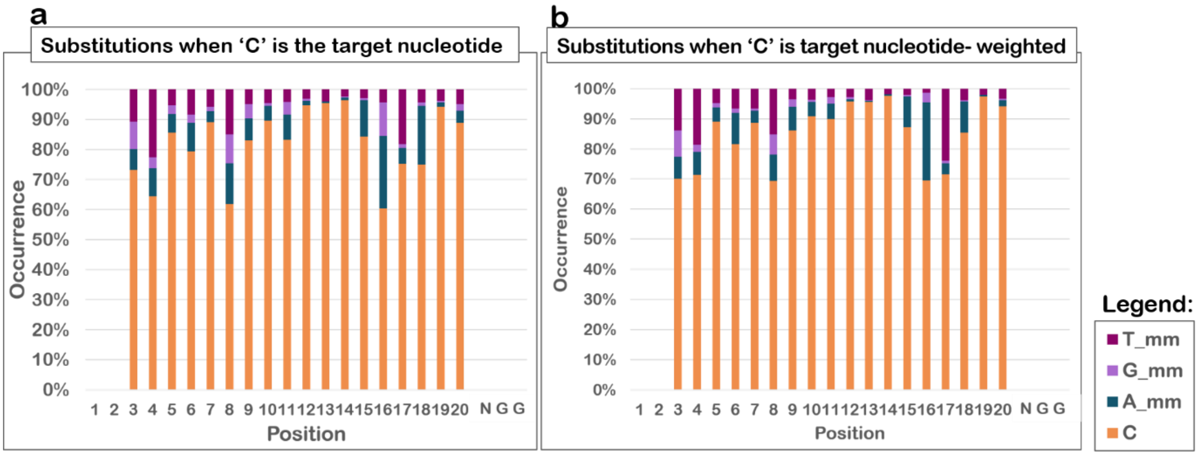

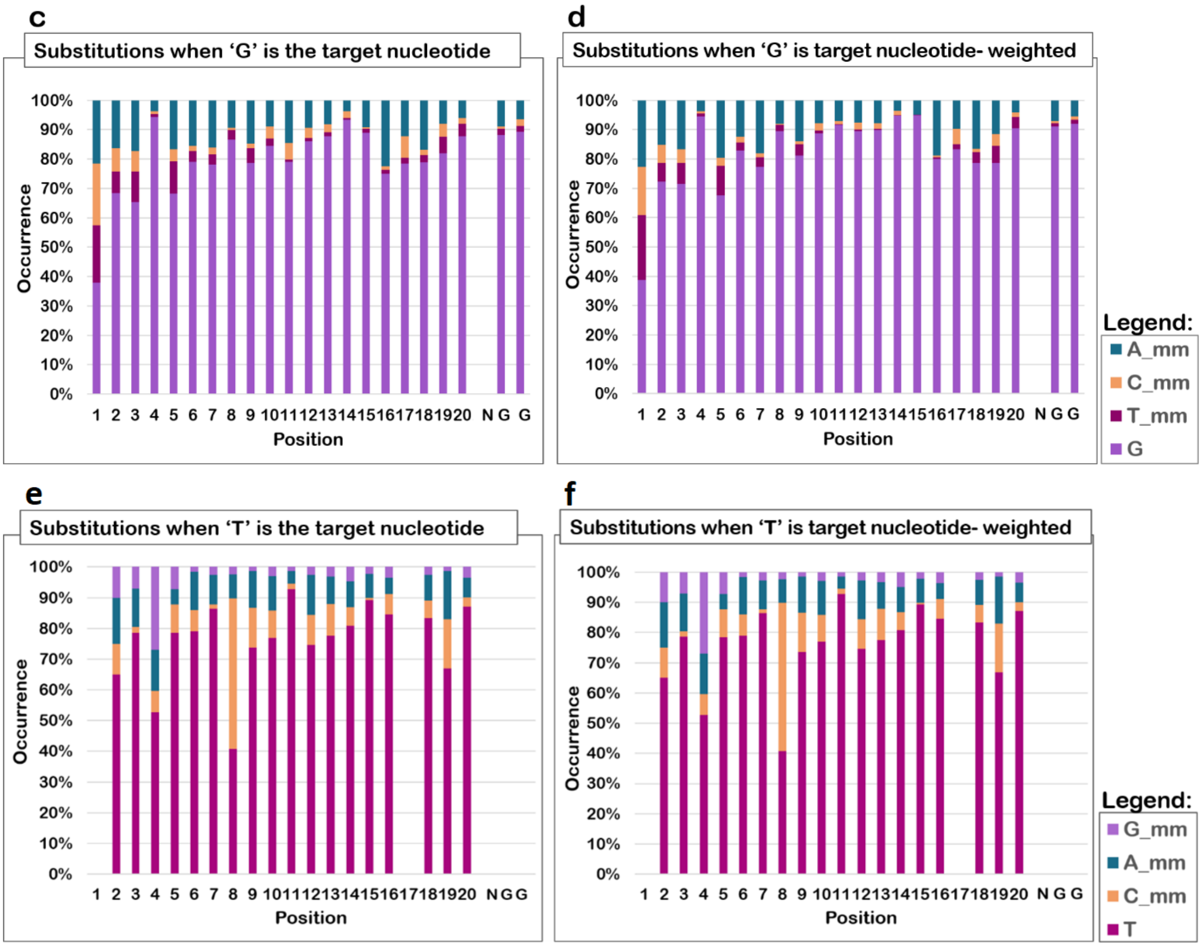

Figure S2. Mismatch tolerance across the length of the guide RNA. The fraction of sites that remain unsubstituted are illustrated, i.e. they do not tolerate mismatches in the positive off-targets evaluated. The plots also illustrate the type of substitutions tolerated in each case when the target nucleotides are 'C' (a) and (b), 'G' (c) and (d), and 'T' (e) and (f). The figures (a), (c) and (e) are for when the number of mismatches are calculated from the sequences once per occurrence. The plots (b), (d) and (f) are for the fraction of mismatches are calculated weighted on the frequency of occurrence in the experiment.

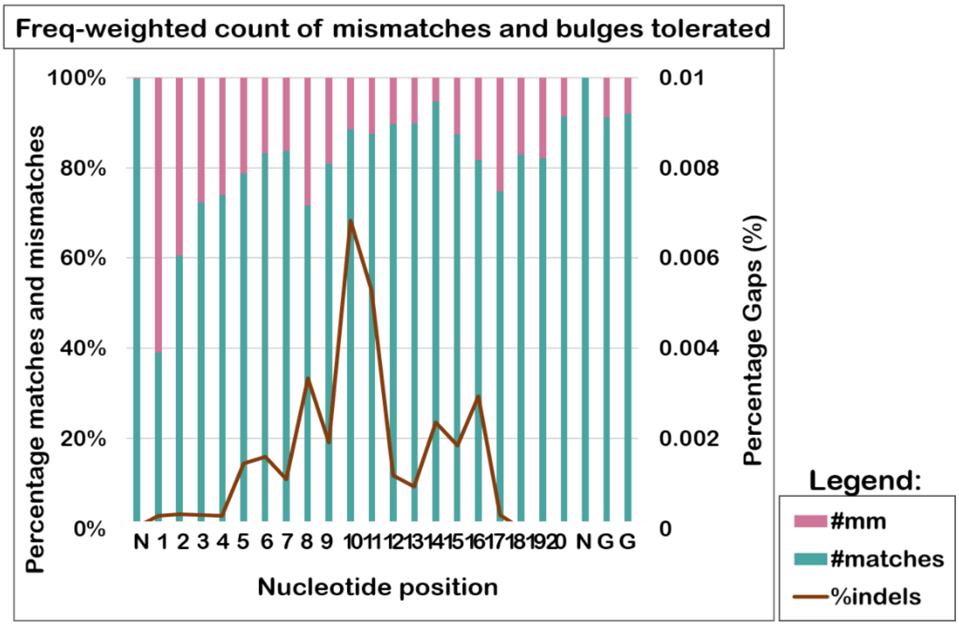

Figure S3. Gap tolerance across the length of the guide RNA. The fraction of gaps at each position among all positive off-target sequences is shown in the figure, weighted by the frequency of occurrence in the experiment. The stacked column plot indicates the percentage of positions at which mismatches are found in the dataset. As can be observed, the gaps are solely clustered towards the centre of the 20-nucleotide sequence while the mismatches are towards the flanks.

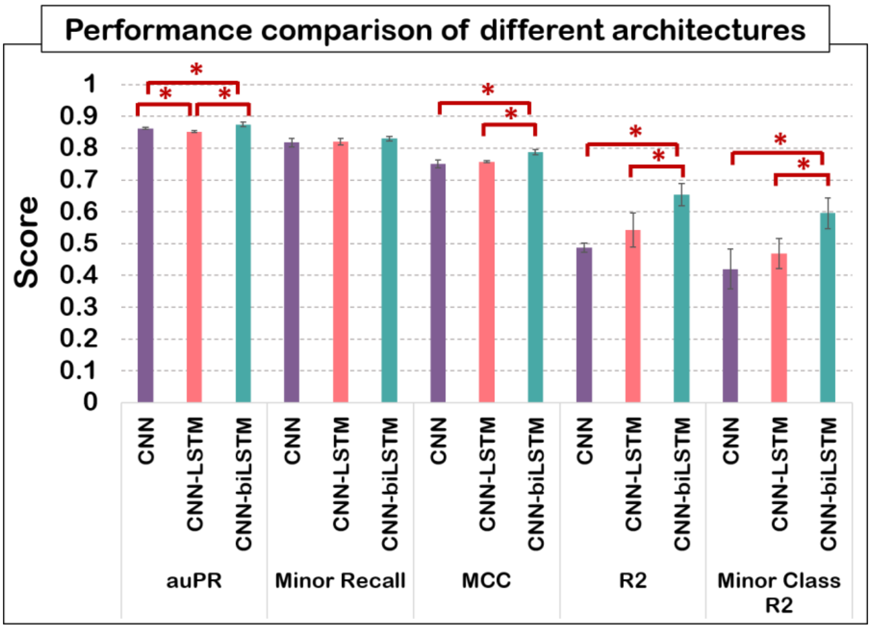

Figure S4. Predictive performance of various neural networks tested. Three architectures were tested for performing simultaneous classification and regression tasks on the dataset- convolutional neural networks (CNN) shown in violet, CNN with an LSTM layer (CNN-LSTM), shown in pink, and CNN with a bidirectional LSTM layer (CNN-biLSTM), shown in green. The three models were optimized, the best performing models were selected and evaluated on independently for 5 runs. The average scores of the area under Precision-Recall curve (auPR), minor class recall, Matthew's correlation coefficient (MCC) was plotted to compare the classification performance and the R2 and minor class R2 were compared for the regression performance. The non-parametric Kruskal-Wallis test was performed to determine if the differences among the scores were significant (* indicates $p<0.01$). The classification performance was comparable, except for the auPR scores, where the CNN-biLSTM outperformed the other architectures. The regression performance of the CNN-biLSTM was better than the other two architectures, hence, was selected for further analyses.

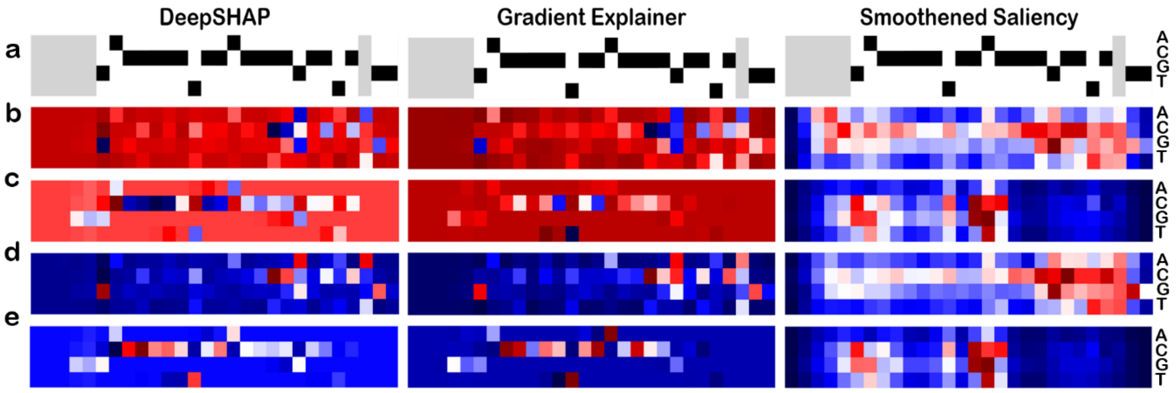

Figure S5. Average Class Activation Maps for the VEGFA2 locus off-targets. Multiple CAMs generated for the off-target sets of the VEGFA2 locus have been averaged using the three techniques of DeepSHAP, Gradient Explainer and smoothened Saliency. (a) The reference target sequence, (b) positive off-target set average CAMs, (c) positive class reference target sequence average CAMs, (d) negative off-target set average CAMs, and (e) negative class reference target sequence for the three algorithms used.

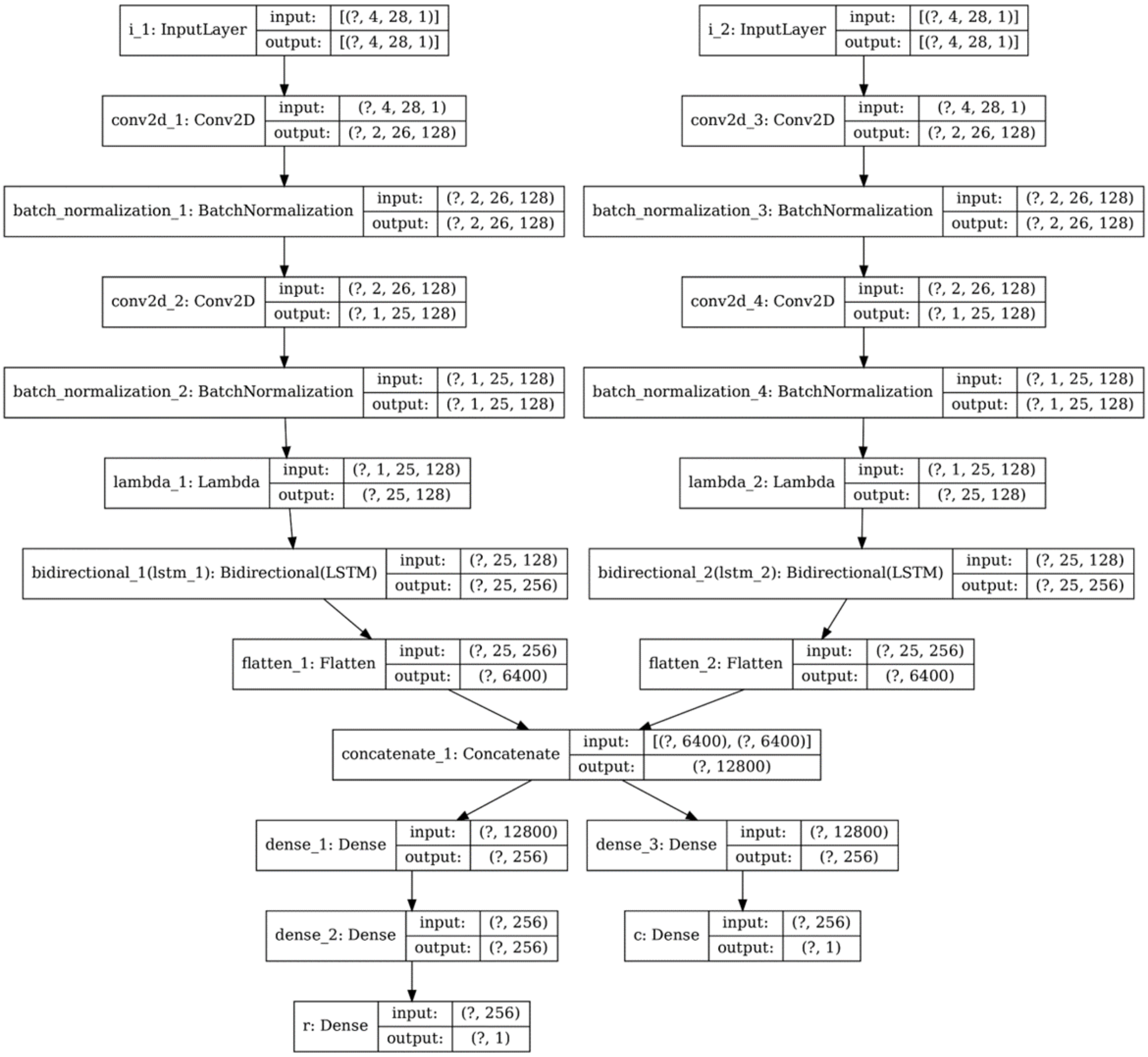

Figure S6. Architecture used for the dual classification and regression tasks. A Siamese-inspired architecture was implemented for the multi-task network. The inputs were one-hot encoded image representations of the target and off-target sequences. The number of nodes in the layers was optimised by grid search.

Table S1. Base-position pairs that tolerate the most mismatches.

Position	Target nucleotide	Sequences with no mismatches (%)	Most substituted nucleotide	Sequences with 'most substituted' mismatch (%)
**3**	G	65.31	A	17.34
**4**	T	52.73	G	27.07
**5**	A	62.76	T	28.99
**6**	T	79.01	A	12.41
**7**	G	78.1	A	16.01
**8**	T	40.71	C	49.08
**9**	A	69.76	T	22.8
**11**	G	79.06	A	14.53
**12**	T	74.61	A	13
**13**	T	77.63	C	10.28
**14**	T	80.3	A	8.33
**15**	A	76.1	G	20.07
**16**	C	60.34	A	24.14
**18**	A	70.39	C	19.49
**19**	T	66.89	C	16.05
**20**	A	75	G	12.01

Table S2. Gap tolerance across the length of guide RNA.

Position	Nucleotide in target	Sequences in which gap is observed (%)
**8**	T	0.008
**8**	C	0.011
**9**	A	0.006
**10**	C	0.007
**10**	G	0.005
**10**	T	0.008
**11**	A	0.007
**16**	T	0.012
**16**	C	0.008

The positions at which notable number of gaps were observed (>0.005% of all match and mismatch occurrences).
